# Adrenal gland volume measurement in depressed patients

**DOI:** 10.1192/j.eurpsy.2025.617

**Published:** 2025-08-26

**Authors:** L. Stepansky, R. Ruppel, L. Sommerfeld, J. Kleiß, K. Türkan, S. Arndt, S. Bickelhaupt, F. Knoll, M. Uder, M. May

**Affiliations:** 1 Institute of Radiology, University Hospital Erlangen; 2 Friedrich-Alexander-University Erlangen-Nürnberg, Erlangen; 3 Department of Radiology, Charité - Universitätsmedizin Berlin, Berlin; 4 Medical Centre for Information and Communication Technology, University Hospital Erlangen; 5 Department Artificial Intelligence in Biomedical Engineering, Friedrich-Alexander-University Erlangen-Nürnberg; 6 Imaging Science Institute, University Hospital Erlangen, Erlangen, Germany

## Abstract

**Introduction:**

Prior studies have shown contradicting results regarding adrenal gland volume (AGV) in depressed patients, with some reporting significant enlargement and others not.

**Objectives:**

The aim of this study was to retrospectively compare CT image segmentations of the adrenal glands in patients with depression to a control group with stringent exclusion criteria to minimize confounding factors.

**Methods:**

We included patients diagnosed with depression (ICD-10: F32/33) who underwent abdominal CT imaging between 2012 and 2022 and did not have any other psychiatric disorders. Diagnoses that could potentially influence AGV were excluded. The resulting 31 depressed patients were compared to a matching control group of 31 patients without depression. The AGV was manually segmented in thin-sliced reconstructions (≤1 mm).

**Results:**

Total AGV in the depressed group was 6.78 (5.19-7.56) cm^3^ compared to 6.90 (5.54-10.05) cm^3^ in the control group. There was no significant difference in AGV between the two groups after adjusting for age, height, and weight. A positive correlation was observed between AGV and height (r=0.41, p<0.001) and weight (r=0.52, p<0.001). Males showed significantly larger AGV than females (p≤0.001), and left AGV was significantly larger than right AGV (p<0.001). Patients within the depressed group who underwent imaging after a suicide attempt showed larger total AGV compared to the control group, though not statistically significant.

**Conclusions:**

AGV is not increased in the well-selected cohort of depressed patients in this study, which contrasts with some previous reports in literature. Further multi-centric studies are required to identify potentially influencing factors such as attempted suicide.

**Disclosure of Interest:**

None Declared

